# B-cell immune repertoire sequencing in tobacco cigarette smoking, vaping, and chronic obstructive pulmonary disease in the COPDGene cohort

**DOI:** 10.3389/fimmu.2025.1508786

**Published:** 2025-05-02

**Authors:** Matthew Moll, Zhonghui Xu, Adel Boueiz, Min Hyung Ryu, Edwin K. Silverman, Michael H. Cho, Craig P. Hersh, Maor Sauler, Francesca Polverino, Gregory L. Kinney, Jeffrey L. Curtis, Laura E. Crotty-Alexander, Christopher Vollmers, Peter J. Castaldi

**Affiliations:** ^1^ Channing Division of Network Medicine, Brigham and Women’s Hospital, Boston, MA, United States; ^2^ Division of Pulmonary and Critical Care Medicine, Brigham and Women’s Hospital, Boston, MA, United States; ^3^ Division of Pulmonary, Critical Care, Sleep and Allergy, Veterans Affairs Boston Healthcare System, West Roxbury, MA, United States; ^4^ Harvard Medical School, Boston, MA, United States; ^5^ Division of Pulmonary, Critical Care, and Sleep Medicine. Yale School of Medicine, New Haven, CT, United States; ^6^ Department of Medicine, Baylor College of Medicine, Houston, TX, United States; ^7^ Department of Epidemiology, Colorado School of Public Health, Aurora, CO, United States; ^8^ Division of Pulmonary and Critical Care, University of Michigan, Ann Arbor, MI, United States; ^9^ Medical Service, Veterans Affairs Ann Arbor Healthcare System, Ann Arbor, MI, United States; ^10^ Pulmonary Critical Care Sleep and Physiology Division, Department of Medicine, University of California, San Diego, San Diego, CA, United States; ^11^ Pulmonary and Critical Care Section, Veterans Affairs San Diego Healthcare System, San Diego, CA, United States; ^12^ Department of Biomolecular Engineering, University of California, Santa Cruz, Santa Cruz, CA, United States; ^13^ Division of General Medicine and Primary Care, Brigham and Women’s Hospital, Boston, MA, United States

**Keywords:** COPD, B cell, immune repertoire, vaping, smoking

## Abstract

**Rationale:**

Cigarette smoking (CS) impairs B-cell function and antibody production, increasing infection risk. The impact of e-cigarette use ('vaping') and combined CS and vaping ('dual-use') on B-cell activity is unclear.

**Objective:**

To examine B-cell receptor sequencing (BCR-seq) profiles associated with CS, vaping, and dual-use.

**Methods:**

BCR-seq was performed on blood RNA samples from 234 participants in the COPDGene study. We assessed multivariable associations of B-cell function measures (immunoglobulin heavy chain (IGH) subclass expression and usage, class-switching, V allele usage, and clonal expansion) with CS, vaping, and dual-use. We adjusted for multiple comparisons using the Benjamini-Hochberg method, identifying significant associations at 5% FDR and suggestive associations at 10% FDR.

**Results:**

Among 234 non-Hispanic white (NHW) and African American (AA) participants, CS and dual-use were significantly positively associated with increased secretory IgA production, with dual-use showing the strongest associations. Dual-use was positively associated with class switching and B-cell clonal expansion, indicating increased B-cell activation, with similar trends in those only smoking or only vaping. The IGHV5-51*01 allele was increased in dual users.

**Conclusions:**

CS and vaping additively enhance B-cell activation, most notably in dual-users. CS and vaping are significantly associated to multiple alterations in B-cell function including increased class switching, clonal expansion, and a shift towards IgA-producing cell populations. These changes could be relevant to response to infection and vaccinations and merit further study.

## Introduction

Use of electronic cigarettes, i.e. vaping, has increased substantially since their introduction to the U.S. market in 2007 (1). Numerous studies have demonstrated that vaping induces inflammatory responses and has adverse health effects (2–6). More precise characterization of the inflammatory effects of vaping may better define its effects on health, both for vaping alone and vaping in conjunction with combustible cigarettes, i.e. dual-use.

B-cells participate in adaptive immunity largely by producing antibodies that protect mucosal surfaces and provide antigen-specific responses to infection. Combustible tobacco cigarette smoking (CS) has adverse effects on B-cells resulting in increased susceptibility to infections (7). Proper B-cell function depends on B-cell activation, a process in which naïve B-cells are activated by exposure to an antigen. This process triggers clonal expansion of B-cell populations with specifically rearranged B-cell receptor (BCR) genes, which encode the specific immunoglobulin (Ig) produced by each clone. In conjunction with T-cell help, these activated clones undergo somatic hypermutation, a prerequisite for affinity maturation, the process by which immunoglobulins are optimized to bind specific antigens. BCR sequencing (BCR-seq) allows for the identification and sequence-specific characterization of B-cells and expanded B-cell clones, providing rich characterization of the B-cell response (8).

We hypothesized that vaping and dual-use alter B-cell function and the ability of B-cells to respond appropriately to antigens through activation and antibody production. To address this question, we performed BCR-seq in 234 participants from the Genetic Epidemiology of COPD (COPDGene) (9) Study, a large study of individuals who currently or previously smoked that is enriched for participants with COPD.

## Methods

### Study population

Written informed consent was obtained from all study participants, and institutional review board approval was obtained at all study centers. The Genetic Epidemiology of COPD (COPDGene) (9) study enrolled 10,198 non-Hispanic white (NHW) and African American (AA) individuals who smoked 10 or more pack-years of cigarettes during their lifetime and who were aged 45–80 at study enrollment. COPDGene is an ongoing longitudinal study with completed enrollment, 5-year, and 10-year visits. At each visit, anthropometric measurements, spirometry, chest computed tomography (CT) imaging, and blood samples were collected. At the 5-year follow up visit, we collected questionnaire data on use of cigarette and e-cigarette products. All data in this paper comes from the 5-year study visit where both blood RNA-seq and vaping data are available.

### Participant selection

Selection of participants for BCR-seq (8) was performed using a stratified random sampling approach as follows. First, all participants with available blood RNA and complete vaping and CS data from COPDGene Phase 2 at the time of participant selection were considered (n=3,601). They were stratified into five groups based on vaping and CS status – never smokers, former combustible cigarette smokers, current cigarette smokers (without current vaping), current vapers (without current cigarette use), and current dual-users (vaping and cigarette use). All participants in the current vaper (n=41) and dual-user group (n=57) with available samples were selected for BCR-seq, and the remaining 136 participants were randomly sampled from the set of available participants in the other CS groups. Participants taking oral corticosteroids were excluded from the analysis as these medications are known to modify B-cell function.

### Definition of key study variables

CS and vaping behavior were ascertained by self-report. Vapers were participants who reported using at least one e-cigarette within the prior week and had a history of smoking tobacco cigarettes, but not within the last 30 days. Current cigarette users reported current smoking with an average of at least one cigarette per day without any e-cigarette use. Dual-users were vapers who also reported current CS, and former cigarette users were defined as those who reported a history of smoking but did not meet criteria for current CS or vaping. In most of the reported analyses, former cigarette users are used as the reference group.

Information on the age, sex, and race of participants was elicited through self-report. For sex, participants were asked if they were male or female. For race, participants were asked if they were NHW, Black or African American, Asian, Pacific Islander, American Indian or Alaska Native, or Other with the option to select multiple categories. By design, inclusion in COPDGene was limited to participants self-identifying as NHW or AA. A separate ethnicity question asked participants if they were Hispanic or Latino. For this study, participants were coded as NHW if they indicated “White” and AA if they indicated “Black or African American.”

In the U.S., COPD primarily develops in the setting of cigarette smoking exposure, and B-cell lymphoid follicles are associated with COPD severity (10–12). Therefore, we examined the association of BCR-seq measures with COPD and COPD-related traits. COPD status was determined by GOLD spirometry grades based on post-bronchodilator spirometry testing where participants were grouped into normal spirometry (FEV1/FVC > 0.7 and FEV1 % predicted > 80%) or GOLD spirometry grade 1, GOLD grade 2-4, or preserved ratio with impaired spirometry (PRISm) (13). For all analyses, the reference group included formerly smoking individuals with normal spirometry. Global Lung Initiative (GLI) race-neutral equations were used to calculate % predicted spirometry values. Computed tomography (CT) imaging measures of emphysema and airway wall thickness were generated by Thirona (https://thirona.eu/) and the following measures were analyzed: % low attenuation area less than -950 Hounsfield units for emphysema and airway wall thickness as % of overall airway volume [wall area percent (14)].

### B-cell receptor sequencing library preparation

Details regarding generation of RNAseq data in COPDGene were previously published (15). Whole blood was collected and stored in PAXgene Blood RNA tubes, and total RNA was extracted using Qiagen PreAnalytiX PAXgene Blood miRNA Kit (Qiagen, Valencia, CA). Sequencing libraries were prepared using 200 ng of total RNA as input following a protocol modified from (8). Additional details regarding library preparation and data processing can be found in the **Supplementary Methods**.

We generated adaptive immune receptor repertoire sequencing data for B-cell receptors (hereafter, ‘BCR-seq’) data using a set of isotype-specific immunoglobulin heavy chain (IGH) constant region primers. Reads were aligned to International Immunogenetics Information System (IMGT) reference germline sequences, and clonal relationships between BCR sequences were inferred using the spectralClones function from the scoper R package contained within the Immcantation suite of software packages (https://immcantation.readthedocs.io/en/stable/about.html). Mutated sequences were defined as sequences that were aligned but differed from the IMGT reference by one or more bases.

Uniquely identified BCR sequences were collapsed by clone and then quantified to represent antibody isotype expression (log 2 counts of the number of unique BCR sequences present within each isotype class) and usage (number of unique BCR sequences present within each isotype class divided by the total number of BCR sequences), B-cell activation measured through class switching (number of unique BCR sequences in the IgA, IgG, and IgE isotypes divided by the total number of BCR sequences), length of the CDR3 region in nucleotides, and the clonal diversity of the B-cell population in each individual as measured by Hill numbers (16). V allele usage was defined as the number of unique and mutated BCR sequences containing a specific V allele (as defined by IGHV genes from the IMGT reference) divided by total number of unique BCR sequences. For each BCR-seq measure, we analyzed only those measures where the isotype or V allele class in question was present at >1% of the total unique sequences for 25% of the participants or more, applying a multiple comparison testing FDR threshold of 0.05, resulting in 10 V segments for analysis. The one exception was measurement of the IgE isotype which was analyzed despite being below this threshold due to its established clinical importance.

### B-cell receptor sequencing measures

BCR sequencing involves sequencing transcripts of the B-cell receptor using a set of primers targeting the Fc-region of the immunoglobin heavy chain (IGH). This provides comprehensive assessment of the BCR repertoire including antibody isotype (IgM, IgD, IgA, IgG, and IgE), V alleles corresponding to the variable region that determines antibody specificity, and clonal expansion and somatic hypermutation of specific B-cell populations. These sequence counts are first collapsed by clone (such that sequences arising from a single B-cell clone are counted as a single occurrence) and summarized into quantitative measures of 1) isotype usage (proportion of antibody transcripts for each isotype within each individual), 2) isotype expression (log2 transformed counts that represent the number of unique B-cells per isotype within each individual), 3) class switching (proportion of class-switched B-cells per individual), 4) V allele usage (proportion of antibody transcripts for each V allele within each individual), 5) CDR3 length by isotype, and 6) B-cell clonal diversity measured by Hill numbers.

### Statistical analysis

We performed analyses in R version >4.0 (www.r-project.org). We assessed normality of continuous variables by visual inspection of histograms. Results are shown as mean ± standard deviation or median [interquartile range], as appropriate. Differences in continuous variables were assessed with Student t-tests or Wilcoxon tests. Categorical variables were compared by ANOVA or Kruskal-Wallis tests, as appropriate. We considered false discovery rate (FDR)-adjusted Benjamini-Hochberg (17) p-values less below 0.05 to be significant and between 0.05 and 0.1 to be suggestive.

For each of the BCR-seq measures, we used univariable analysis and multivariable regression to examine the association between each of these measures with the primary outcomes of CS, vaping, and dual-use. Further, we examined associations to secondary outcomes, including age, sex, and race as well as COPD affection status (GOLD 2-4), CT emphysema measures [ % low attenuation area (LAA) < -950 Hounsfeld units (HU) (14)], and CT airway wall thickness [wall area % (14)]. Age was calculated from the study date and the date of birth. Sex was determined by the concordance of self-report and XX or XY status. Race was based on self-report. Principal components of genetic similarity were calculated using Eigensoft 3.0, as previously published (18).

For analyses of smoking/vaping, demographic variables, and GOLD spirometry grade, multivariable models included the following covariates: age, sex, self-identified race, vaping/smoking behavior, GOLD grade, pack-years of smoking, and inhaled corticosteroid use. For CT imaging measures, models were additionally adjusted for CT scanner model. To visualize the results, we constructed violin plots and heatmaps.

Sensitivity analyses were performed for the significant associations observed with smoking/vaping status and self-reported race adjusting for self-reported income level and social deprivation index, a measure of area-level deprivation (19), and principal components of genetic similarity. We additionally performed interaction analyses between self-reported race and smoking/vaping variables by including the main effects and cross-product interaction terms in a regression model.

## Results

### Characteristics of study participants

**Figure 1:**
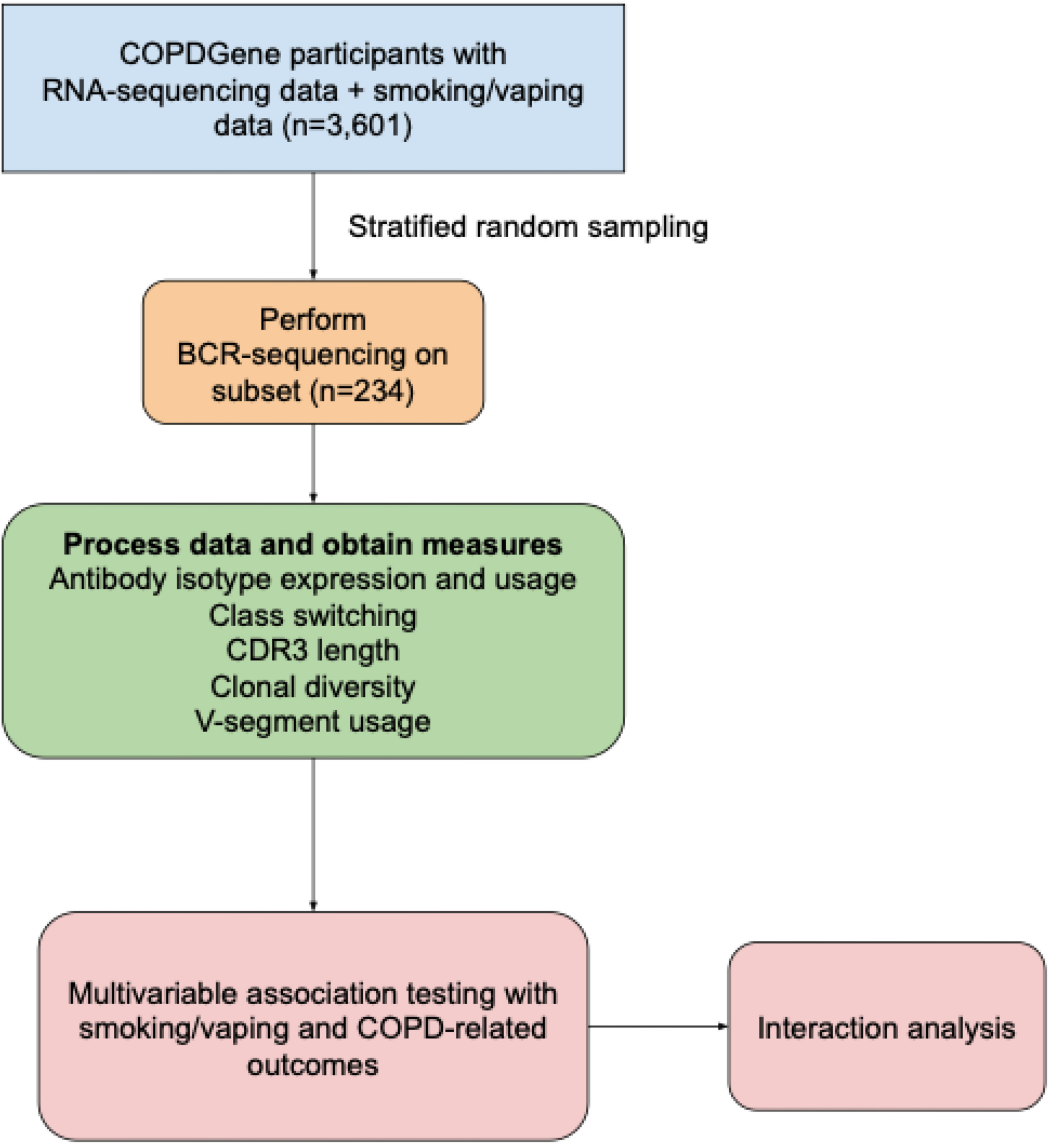
Schematic of study design. COPDGene, Genetic Epidemiology of COPD study; BCR, B-cell receptor; COPD, chronic obstructive pulmonary disease.

A schematic of our study design is shown in **Figure 1**. We included 234 COPDGene NHW and AA participants with smoking/vaping and BCR-seq data, and a table of their characteristics is shown in **Table 1**. Compared to other groups, dual-users were more likely to be younger, NHW, have more pack-years of cigarette smoking (CS), lower FEV_1_ % predicted, and thicker airway walls. Compared to dual-users, individuals who only vaped were slightly older, were less likely to be male, had similar pack-years of smoking, but had higher FEV_1_ % predicted, and more quantitative emphysema (% LAA < -950 HU). To evaluate for bias with respect to which individuals had available RNA-seq data in the overall COPDGene cohort, we compared characteristics and found similar rates of smoking and vaping, though fewer African Americans in the group had available RNA-seq data (**Table 2**).

**Table 1:** Characteristics of study participants.

	Former smoker	Never	Vaping	Cigarette smoking	Dual	Overall	p-value
	(N=44)	(N=41)	(N=41)	(N=51)	(N=57)	(N=234)	
age	69.6 (7.42)	65.3 (9.96)	64.4 (6.56)	62.0 (7.21)	61.3 (6.47)	64.2 (8.04)	<0.001
sex							
female	23 (52.3%)	29 (70.7%)	26 (63.4%)	31 (60.8%)	29 (50.9%)	138 (59.0%)	0.403
male	21 (47.7%)	12 (29.3%)	15 (36.6%)	20 (39.2%)	28 (49.1%)	96 (41.0%)	
race							
AA	6 (13.6%)	2 (4.9%)	4 (9.8%)	28 (54.9%)	15 (26.3%)	55 (23.5%)	<0.001
NHW	38 (86.4%)	39 (95.1%)	37 (90.2%)	23 (45.1%)	42 (73.7%)	179 (76.5%)	
cigarette pack-years	43.0 (23.3)	0 (0)	52.2 (24.6)	48.5 (27.8)	52.9 (25.3)	40.7 (29.8)	<0.001
GOLD spirometry grade							
Normal spirometry	15 (34.1%)	39 (95.1%)	19 (46.3%)	22 (43.1%)	19 (33.3%)	114 (48.7%)	<0.001
GOLD 1	6 (13.6%)	0 (0%)	5 (12.2%)	3 (5.9%)	6 (10.5%)	20 (8.5%)	
GOLD 2,3,4	21 (47.7%)	1 (2.4%)	13 (31.7%)	20 (39.2%)	29 (50.9%)	84 (35.9%)	
PRISm	2 (4.5%)	1 (2.4%)	4 (9.8%)	6 (11.8%)	3 (5.3%)	16 (6.8%)	
FEV1, % of predicted	75.3 (27.7)	74.1 (26.8)	107 (13.2)	72.8 (21.1)	82.0 (24.9)	81.3 (26.5)	<0.001
FEV1/FVC	0.633 (0.149)	0.795 (0.0479)	0.672 (0.142)	0.688 (0.141)	0.642 (0.158)	0.682 (0.146)	<0.001
CT imaging							
CT imaging emphysema	6.30 (7.55)	1.24 (1.44)	3.43 (4.90)	3.43 (7.13)	3.10 (5.76)	3.45 (5.99)	0.002
CT airway wall thickness	51.1 (6.34)	42.7 (4.81)	48.4 (8.79)	52.6 (8.97)	52.6 (9.14)	49.8 (8.67)	<0.001

Values are mean (standard deviation) for continuous variables and N (%) for categorical variables. CT emphysema measurement is % low attenuation area <-950 Hounsfield units. CT airway wall thickness is defined as (area of segmental airway walls / overall airway area). FEV1, forced expiratory volume in 1 second; FEV1/FVC, FEV1/forced vital capacity; PRISm, preserved ratio with impaired spirometry; AA, African American; NHW, non-Hispanic white; CT, computed tomography. Global Lung Initiative (GLI) race-neutral equations were used to calculate % predicted values. P-values test differences across all groups [analysis of variance (ANOVA)].

**Table 2:** Characteristics of COPDGene Visit 2 participants with vaping data comparing those with and without RNA-seq data.

Characteristic	No RNA-seq	RNA-seq	p
n	1419	3192	
Age in years (mean (SD))	64.30 (8.61)	65.29 (8.73)	<0.001
Sex, female (No. %)	725 (51.1)	1574 (49.3)	0.278
Race, African American (No. %)	531 (37.4)	845 (26.5)	<0.001
FEV1 % predicted (mean (SD))	78.45 (24.74)	80.84 (25.10)	0.003
FEV1/FVC (mean (SD))	0.69 (0.15)	0.69 (0.14)	0.95
Pack-years of smoking (mean (SD))	38.75 (25.45)	41.42 (25.55)	0.001
Current smoking status (No. %)	567 (40.0)	1149 (36.0)	0.012
Ever vaping status (No. %)	157 (11.1)	358 (11.2)	0.92
Current vaping status (No. %)	52 (33.3)	109 (31.0)	0.67

### Associations between BCR-seq measures and cigarette smoking, vaping, and dual-use

**Figure 2:**
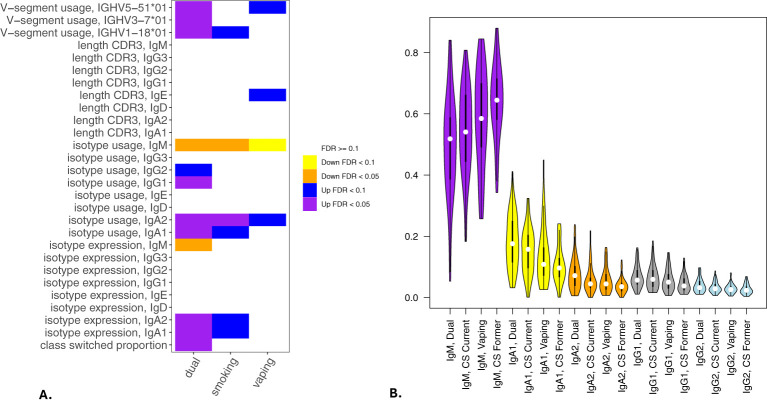
Associations of BCR measures with vaping and cigarette smoking. Significant associations between BCR measures and vaping, cigarette smoking, and dual-use from multivariable models analyzing class switching, isotype expression and usage, V allele usage, and CDR3 length (in nucleotides) are shown in **(A)**. **(B)** shows IgM, IgA and IgG isotype usage among participants engaged in current smoking, vaping, or dual-use with former smokers included for comparison. Significance is assessed by t-tests. *p<=0.05, **p<=0.005, ns p>0.05.

**Figure 3:**
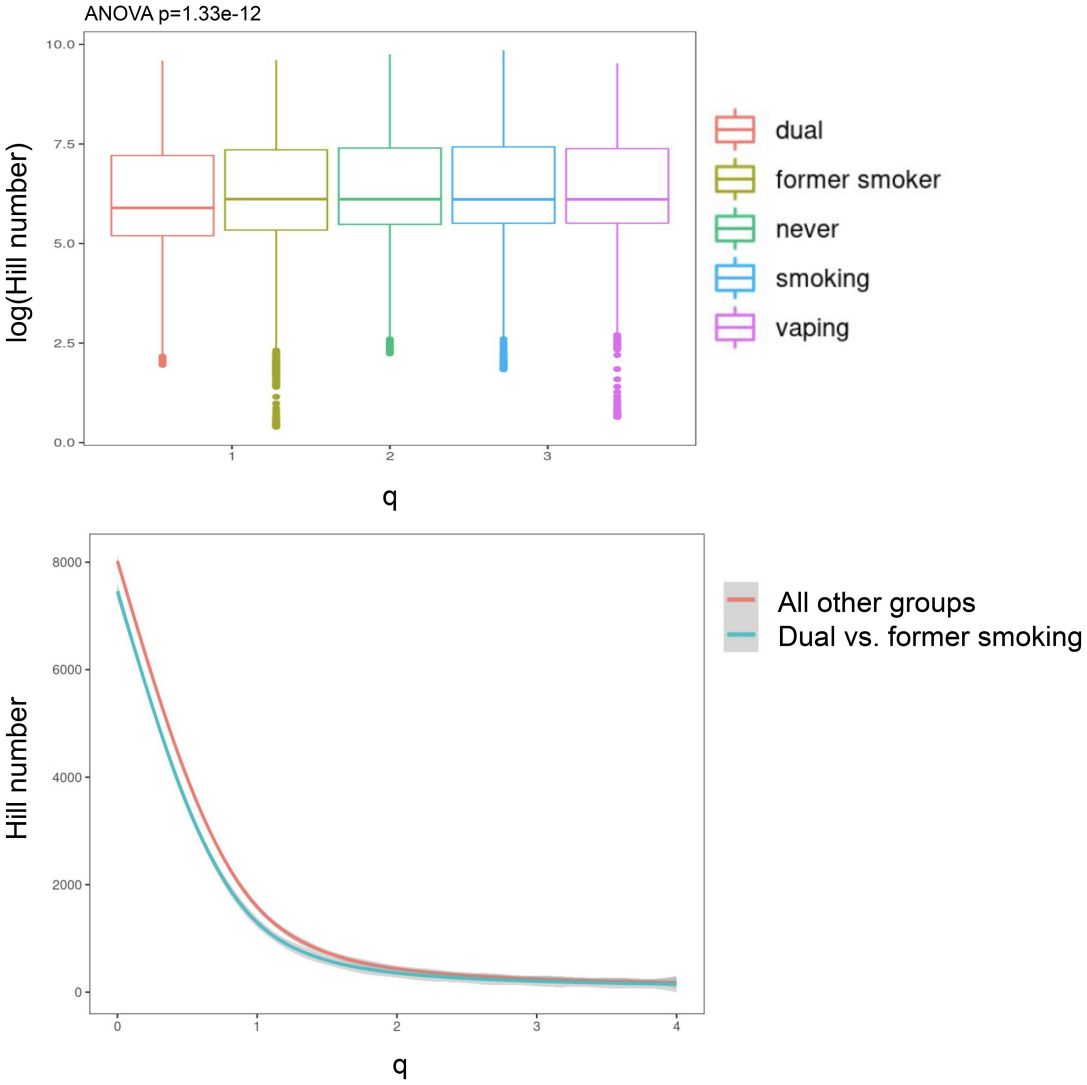
Hill biodiversity numbers show less antibody diversity (and more clonal expansion) in dual users relative to other smoking/vaping groups. The top panel is a boxplot showing that the dual users (i.e., vaping + smoking) have lower log-Hill values than other groups. In the bottom panel, we collapsed all other groups and compared the hill numbers in a continuous fashion over a range of q-values.

Significant (q-value < 0.05) and suggestive (q-value <0.1) associations for vaping, CS, and dual-use are shown in– **Table 3**, while complete model results are shown in **Tables 4**–**8**. Overall, we observed that the most pronounced changes in antibody production were associated with dual-use. Specifically, dual-use resulted in a shift in isotype usage towards IgA, IgG1 and IgG2, and away from IgM (**Figure 2A**). It was also associated with increased class switching suggestive of B-cell activation (**Figure 2B**) and increased usage of specific V alleles, most notably IGHV5-51*01, IGHV3-1*01, and IGHV1-18*01. Hill biodiversity analysis also demonstrated reduced antibody diversity for participants engaged in dual-use (**Figure 3**), suggesting that there is a greater amount of B-cell clonal expansion in this group. Since CS status is often associated with socioeconomic variables, we tested these associations after adjusting for income level and social deprivation index, a composite measure of area-level deprivation, which had minimal effect on the significance of these associations (**Table 9**).

**Table 3:** Significant BCR associations to vaping, cigarette smoking, and dual-use.

BCR measure	Dual-use			Cigarette smoking			Vaping		
	β (se)	P-value	q-value	β (se)	P-value	q-value	β (se)	P-value	q-value
IgA1 expression	0.573 (0.18)	1.70E-03	2.86E-02	0.49 (0.189)	1.02E-02	7.54E-02	0.231 (0.186)	2.16E-01	5.25E-01
IgA2 expression	0.731 (0.203)	3.83E-04	9.64E-03	0.597 (0.212)	5.27E-03	5.53E-02	0.455 (0.209)	3.02E-02	1.49E-01
IgM expression	-0.392 (0.135)	4.07E-03	4.46E-02	-0.096 (0.142)	4.96E-01	7.79E-01	-0.262 (0.139)	6.13E-02	2.27E-01
IgA1 usage	0.075 (0.017)	1.14e-05	5.84E-04	0.047 (0.017)	7.08E-03	6.15E-02	0.037 (0.017)	3.45E-02	1.64E-01
IgA2 usage	0.041 (0.008)	2.57e-06	2.16E-04	0.026 (0.009)	3.99E-03	4.46E-02	0.024 (0.009)	6.19E-03	6.00E-02
IgG1 usage	0.021 (0.007)	2.37E-03	3.43E-02	0.013 (0.007)	6.96E-02	2.47E-01	0.015 (0.007)	3.76E-02	1.67E-01
IgG2 usage	0.012 (0.004)	5.49E-03	5.54E-02	0.004 (0.004)	3.19E-01	6.21E-01	0.005 (0.004)	2.40E-01	5.45E-01
IgM usage	-0.148 (0.03)	2.14e-06	2.16E-04	-0.095 (0.032)	3.21E-03	3.85E-02	-0.082 (0.031)	9.79E-03	7.48E-02
Class switching	0.111 (0.027)	5.36e-05	2.22E-03	0.064 (0.028)	2.36E-02	1.29E-01	0.05 (0.028)	7.42E-02	2.52E-01
IGHV1-18*01 class switching	0.005 (0.001)	4.89E-04	1.03E-02	0.004 (0.001)	9.35E-03	7.36E-02	0.004 (0.001)	1.62E-02	1.02E-01
IGHV3-7*01 class switching	0.006 (0.002)	3.22E-04	9.00E-03	0.003 (0.002)	5.31E-02	2.05E-01	0.002 (0.002)	2.82E-01	6.00E-01
IGHV5-51*01 class switching	0.009 (0.002)	6.16e-05	2.22E-03	0.005 (0.002)	1.79E-02	1.10E-01	0.006 (0.002)	1.31E-02	8.94E-02
IgE CDR3 Length	0.439 (2.958)	8.82E-01	9.70E-01	2.849 (3.03)	3.49E-01	6.33E-01	8.151 (3.261)	1.39E-02	9.23E-02

Count values are log2 of unique BCR RNA sequence count. Usage is the proportion of all BCR RNA sequences falling into either a specific isotype or V allele category (calculated separately for isotypes and V alleles). Class switching proportion is the proportion of all BCR RNA sequences that belong to IgA, IgG, or IgE isotypes and have evidence of somatic hypermutation (>1 mutation relative to the IMGT reference database). CDR3 length is the length of the CDR3 sequence in nucleotides. Q-value is calculated using the Benjamini-Hochberg method.

**Table 4:** Regression models for isotype usage with independent variables of interest.

Dependent Variable	Independent Variables	beta (se)	P-value	q-value
IGA1 usage	age	0 (0.001)	0.62	0.86
	sex	0.01 (0.01)	0.35	0.63
	race	-0.008 (0.014)	0.55	0.8
	dual use	0.075 (0.017)	1.10E-05	0.00058
	smoking	0.047 (0.017)	0.0071	0.062
	vaping	0.037 (0.017)	0.034	0.16
	GOLD 1 vs 0	0.022 (0.02)	0.26	0.58
	GOLD 2–4 vs 0	0.022 (0.013)	0.088	0.28
	PRISm vs 0	-0.019 (0.021)	0.38	0.66
IGA2 usage	age	0 (0)	0.54	0.8
	sex	0.016 (0.005)	0.0029	0.037
	race	-0.003 (0.007)	0.66	0.87
	dual use	0.041 (0.008)	2.60E-06	0.00022
	smoking	0.026 (0.009)	0.004	0.045
	vaping	0.024 (0.009)	0.0062	0.06
	GOLD 1 vs 0	0.009 (0.01)	0.37	0.65
	GOLD 2–4 vs 0	0.014 (0.007)	0.038	0.17
	PRISm vs 0	-0.011 (0.011)	0.31	0.61
IGD usage	age	0 (0)	0.84	0.95
	sex	0.005 (0.007)	0.46	0.76
	race	0.001 (0.009)	0.9	0.97
	dual use	-0.015 (0.011)	0.16	0.41
	smoking	0 (0.011)	0.99	0.99
	vaping	-0.002 (0.011)	0.85	0.95
	GOLD 1 vs 0	-0.002 (0.013)	0.85	0.95
	GOLD 2–4 vs 0	-0.003 (0.008)	0.68	0.87
	PRISm vs 0	0.009 (0.014)	0.52	0.79
IGE usage	age	0 (0)	0.4	0.68
	sex	0 (0)	0.021	0.12
	race	0 (0)	0.05	0.2
	dual use	0 (0)	0.29	0.6
	smoking	0 (0)	0.57	0.82
	vaping	0 (0)	0.93	0.99
	GOLD 1 vs 0	0 (0)	0.43	0.73
	GOLD 2–4 vs 0	0 (0)	0.054	0.2
	PRISm vs 0	0 (0)	0.89	0.97
IGG1 usage	age	0 (0)	0.12	0.35
	sex	-0.003 (0.004)	0.54	0.8
	race	-0.025 (0.006)	1.20E-05	0.00058
	dual use	0.021 (0.007)	0.0024	0.034
	smoking	0.013 (0.007)	0.07	0.25
	vaping	0.015 (0.007)	0.038	0.17
	GOLD 1 vs 0	-0.006 (0.008)	0.47	0.76
	GOLD 2–4 vs 0	0.002 (0.005)	0.69	0.87
	PRISm vs 0	-0.009 (0.009)	0.34	0.63
IGG2 usage	age	0 (0)	0.69	0.87
	sex	0.005 (0.003)	0.036	0.17
	race	-0.008 (0.003)	0.023	0.13
	dual use	0.012 (0.004)	0.0055	0.055
	smoking	0.004 (0.004)	0.32	0.62
	vaping	0.005 (0.004)	0.24	0.54
	GOLD 1 vs 0	0.001 (0.005)	0.79	0.91
	GOLD 2–4 vs 0	0.004 (0.003)	0.23	0.53
	PRISm vs 0	0 (0.005)	0.96	0.99
IGG3 usage	age	0 (0)	0.98	0.99
	sex	0 (0.001)	0.61	0.85
	race	-0.004 (0.001)	0.00013	0.0042
	dual use	0.003 (0.001)	0.04	0.17
	smoking	0 (0.001)	0.93	0.99
	vaping	0.001 (0.001)	0.44	0.74
	GOLD 1 vs 0	0.002 (0.002)	0.26	0.58
	GOLD 2–4 vs 0	0.002 (0.001)	0.051	0.2
	PRISm vs 0	0 (0.002)	0.94	0.99
IGM usage	age	-0.002 (0.001)	0.23	0.53
	sex	-0.035 (0.019)	0.066	0.24
	race	0.063 (0.025)	0.011	0.082
	dual use	-0.148 (0.03)	2.10E-06	0.00022
	smoking	-0.095 (0.032)	0.0032	0.039
	vaping	-0.082 (0.031)	0.0098	0.075
	GOLD 1 vs 0	-0.022 (0.036)	0.54	0.8
	GOLD 2–4 vs 0	-0.04 (0.024)	0.093	0.29
	PRISm vs 0	0.035 (0.039)	0.37	0.65

Usage is the proportion of all BCR RNA sequences falling into either a specific isotype or V allele category (calculated separately for isotypes and V alleles). Sex coded as (0=female, 1=male). Race coded as (0=self-reported Black race, 1=self-reported White race). PrISM, Preserved ratio impaired spirometry.

**Table 5:** Regression models for isotype expression with independent variables of interest.

Dependent Variable	Independent Variables	beta (se)	P-value	q-value
IGA1 expression	age	-0.008 (0.008)	0.29	0.6
	sex	0 (0.113)	1	1
	race	0.003 (0.147)	0.98	0.99
	dual use	0.573 (0.18)	0.0017	0.029
	smoking	0.49 (0.189)	0.01	0.075
	vaping	0.231 (0.186)	0.22	0.53
	GOLD 1 vs 0	-0.008 (0.214)	0.97	0.99
	GOLD 2–4 vs 0	0.063 (0.14)	0.65	0.87
	PRISm vs 0	-0.449 (0.231)	0.053	0.2
IGA2 expression	age	-0.004 (0.009)	0.65	0.87
	sex	0.233 (0.127)	0.069	0.25
	race	0.006 (0.166)	0.97	0.99
	dual use	0.731 (0.203)	0.00038	0.0096
	smoking	0.597 (0.212)	0.0053	0.055
	vaping	0.455 (0.209)	0.03	0.15
	GOLD 1 vs 0	-0.113 (0.239)	0.64	0.87
	GOLD 2–4 vs 0	0.082 (0.158)	0.6	0.85
	PRISm vs 0	-0.553 (0.258)	0.033	0.16
IGD expression	age	-0.013 (0.006)	0.052	0.2
	sex	-0.083 (0.094)	0.38	0.65
	race	0.146 (0.121)	0.23	0.53
	dual use	-0.225 (0.149)	0.13	0.37
	smoking	0.043 (0.156)	0.78	0.91
	vaping	-0.131 (0.153)	0.39	0.67
	GOLD 1 vs 0	0.101 (0.176)	0.57	0.81
	GOLD 2–4 vs 0	-0.137 (0.116)	0.24	0.54
	PRISm vs 0	0.014 (0.19)	0.94	0.99
IGE expression	age	0.005 (0.012)	0.71	0.87
	sex	-0.275 (0.176)	0.12	0.35
	race	-0.569 (0.215)	0.0093	0.074
	dual use	-0.097 (0.266)	0.72	0.87
	smoking	0.193 (0.273)	0.48	0.77
	vaping	0.114 (0.293)	0.7	0.87
	GOLD 1 vs 0	0.304 (0.417)	0.47	0.76
	GOLD 2–4 vs 0	0.227 (0.202)	0.26	0.58
	PRISm vs 0	-0.032 (0.379)	0.93	0.99
IGG1 expression	age	-0.006 (0.007)	0.33	0.63
	sex	-0.104 (0.097)	0.29	0.6
	race	-0.384 (0.126)	0.0026	0.034
	dual use	0.341 (0.154)	0.027	0.14
	smoking	0.292 (0.162)	0.073	0.25
	vaping	0.164 (0.159)	0.3	0.61
	GOLD 1 vs 0	0.088 (0.182)	0.63	0.86
	GOLD 2–4 vs 0	-0.048 (0.12)	0.69	0.87
	PRISm vs 0	-0.189 (0.197)	0.34	0.63
IGG2 expression	age	-0.012 (0.007)	0.073	0.25
	sex	0.091 (0.1)	0.36	0.65
	race	-0.186 (0.13)	0.15	0.41
	dual use	0.272 (0.16)	0.09	0.29
	smoking	0.246 (0.166)	0.14	0.38
	vaping	0.092 (0.163)	0.58	0.82
	GOLD 1 vs 0	0.176 (0.188)	0.35	0.63
	GOLD 2–4 vs 0	0.029 (0.124)	0.81	0.93
	PRISm vs 0	-0.079 (0.202)	0.7	0.87
IGG3 expression	age	-0.01 (0.008)	0.21	0.52
	sex	-0.121 (0.11)	0.27	0.59
	race	-0.5 (0.143)	0.00056	0.011
	dual use	0.331 (0.175)	0.059	0.22
	smoking	0.179 (0.184)	0.33	0.63
	vaping	0.063 (0.18)	0.72	0.87
	GOLD 1 vs 0	0.276 (0.207)	0.18	0.46
	GOLD 2–4 vs 0	0.089 (0.137)	0.52	0.79
	PRISm vs 0	-0.277 (0.223)	0.22	0.53
IGM expression	age	-0.015 (0.006)	0.0087	0.073
	sex	-0.177 (0.085)	0.038	0.17
	race	0.196 (0.11)	0.075	0.25
	dual use	-0.392 (0.135)	0.0041	0.045
	smoking	-0.096 (0.142)	0.5	0.78
	vaping	-0.262 (0.139)	0.061	0.23
	GOLD 1 vs 0	0.057 (0.16)	0.72	0.87
	GOLD 2–4 vs 0	-0.169 (0.105)	0.11	0.32
	PRISm vs 0	-0.009 (0.173)	0.96	0.99

Count values are log2 of unique BCR RNA sequence count. Sex coded as (0=female, 1=male). Race coded as (0=self-reported Black race, 1=self-reported White race). PrISM, Preserved ratio impaired spirometry.

**Table 6:** Regression models for V allele usage with independent variables of interest.

Dependent Variable	Independent Variables	beta (se)	P-value	q-value
IGHV1-18*01 usage	age	0 (0)	0.51	0.78
	sex	0.001 (0.001)	0.28	0.6
	race	-0.003 (0.001)	0.02	0.12
	dual use	0.005 (0.001)	0.00049	0.01
	smoking	0.004 (0.001)	0.0093	0.074
	vaping	0.004 (0.001)	0.016	0.1
	GOLD 1 vs 0	0.001 (0.002)	0.37	0.65
	GOLD 2–4 vs 0	0.001 (0.001)	0.24	0.55
	PRISm vs 0	-0.001 (0.002)	0.5	0.78
IGHV3-7*01 usage	age	0 (0)	0.11	0.32
	sex	0.003 (0.001)	0.013	0.089
	race	-0.004 (0.001)	0.0018	0.029
	dual use	0.006 (0.002)	0.00032	0.009
	smoking	0.003 (0.002)	0.053	0.2
	vaping	0.002 (0.002)	0.28	0.6
	GOLD 1 vs 0	0 (0.002)	0.87	0.96
	GOLD 2–4 vs 0	-0.001 (0.001)	0.55	0.8
	PRISm vs 0	0 (0.002)	0.92	0.99
IGHV5-51*01 usage	age	0 (0)	0.35	0.63
	sex	0.003 (0.001)	0.015	0.098
	race	-0.001 (0.002)	0.68	0.87
	dual use	0.009 (0.002)	6.20E-05	0.0022
	smoking	0.005 (0.002)	0.018	0.11
	vaping	0.006 (0.002)	0.013	0.089
	GOLD 1 vs 0	-0.002 (0.003)	0.46	0.76
	GOLD 2–4 vs 0	0.004 (0.002)	0.03	0.15
	PRISm vs 0	-0.002 (0.003)	0.55	0.8

Usage is the proportion of all BCR RNA sequences falling into either a specific isotype or V allele category (calculated separately for isotypes and V alleles). Sex coded as (0=female, 1=male). Race coded as (0=self-reported Black race, 1=self-reported White race). PrISM, Preserved ratio impaired spirometry. V alleles where 75th percentile of usage is >0.01 were analyzed.

**Table 7:** Regression model for class switching with independent variables of interest.

Dependent Variable	Independent Variables	beta (se)	P-value	q-value
class switching	age	0.001 (0.001)	0.47	0.76
	sex	0.033 (0.017)	0.05	0.2
	race	-0.059 (0.022)	0.007	0.062
	dual use	0.111 (0.027)	5.40E-05	0.0022
	smoking	0.064 (0.028)	0.024	0.13
	vaping	0.05 (0.028)	0.074	0.25
	GOLD 1 vs 0	0.011 (0.032)	0.72	0.87
	GOLD 2–4 vs 0	0.028 (0.021)	0.18	0.46
	PRISm vs 0	-0.013 (0.034)	0.71	0.87

Class switching proportion is the proportion of all BCR RNA sequences that belong to IgA, IgG, or IgE isotypes and have evidence of somatic hypermutation (>1 mutation relative to the IMGT reference database). Sex coded as (0=female, 1=male). Race coded as (0=self-reported Black race, 1=self-reported White race). PrISM, Preserved ratio impaired spirometry.

**Table 8:** Regression models for CDR3 length with independent variables of interest.

Dependent Variable	Independent Variables	beta (se)	P-value	q-value
IGA1 CDR3 Length	age	-0.003 (0.01)	0.76	0.89
	sex	-0.247 (0.144)	0.087	0.28
	race	0.645 (0.186)	0.00061	0.011
	dual use	0.161 (0.228)	0.48	0.77
	smoking	0.046 (0.239)	0.85	0.95
	vaping	-0.012 (0.236)	0.96	0.99
	GOLD 1 vs 0	0.281 (0.27)	0.3	0.6
	GOLD 2–4 vs 0	-0.049 (0.178)	0.78	0.91
	PRISm vs 0	0.167 (0.292)	0.57	0.81
IGA2 CDR3 Length	age	-0.016 (0.013)	0.21	0.52
	sex	0.006 (0.191)	0.97	0.99
	race	-0.095 (0.247)	0.7	0.87
	dual use	0.5 (0.305)	0.1	0.31
	smoking	0.471 (0.321)	0.14	0.39
	vaping	0.385 (0.316)	0.22	0.53
	GOLD 1 vs 0	0.79 (0.36)	0.029	0.15
	GOLD 2–4 vs 0	0.24 (0.236)	0.31	0.61
	PRISm vs 0	0.114 (0.399)	0.78	0.91
IGD CDR3 Length	age	-0.006 (0.014)	0.68	0.87
	sex	-0.466 (0.199)	0.02	0.12
	race	0.914 (0.257)	0.00046	0.01
	dual use	0.118 (0.316)	0.71	0.87
	smoking	-0.137 (0.331)	0.68	0.87
	vaping	-0.147 (0.326)	0.65	0.87
	GOLD 1 vs 0	0.133 (0.374)	0.72	0.87
	GOLD 2–4 vs 0	-0.403 (0.246)	0.1	0.31
	PRISm vs 0	0.131 (0.404)	0.75	0.89
IGE CDR3 Length	age	-0.116 (0.133)	0.39	0.66
	sex	0.412 (1.964)	0.83	0.95
	race	-2.208 (2.386)	0.36	0.64
	dual use	0.439 (2.958)	0.88	0.97
	smoking	2.849 (3.03)	0.35	0.63
	vaping	8.151 (3.261)	0.014	0.092
	GOLD 1 vs 0	-1.249 (4.641)	0.79	0.91
	GOLD 2–4 vs 0	0.107 (2.245)	0.96	0.99
	PRISm vs 0	-4.16 (4.22)	0.33	0.63
IGG1 CDR3 Length	age	-0.009 (0.013)	0.48	0.76
	sex	0.068 (0.194)	0.73	0.87
	race	1.273 (0.25)	7.90E-07	2.00E-04
	dual use	0.039 (0.309)	0.9	0.97
	smoking	0.025 (0.323)	0.94	0.99
	vaping	-0.622 (0.318)	0.052	0.2
	GOLD 1 vs 0	0.381 (0.364)	0.3	0.6
	GOLD 2–4 vs 0	-0.348 (0.24)	0.15	0.4
	PRISm vs 0	-0.48 (0.394)	0.22	0.53
IGG2 CDR3 Length	age	-0.01 (0.015)	0.5	0.78
	sex	-0.362 (0.221)	0.1	0.31
	race	-0.043 (0.286)	0.88	0.97
	dual use	0.006 (0.352)	0.99	0.99
	smoking	0.097 (0.369)	0.79	0.91
	vaping	-0.136 (0.363)	0.71	0.87
	GOLD 1 vs 0	0.149 (0.416)	0.72	0.87
	GOLD 2–4 vs 0	-0.403 (0.274)	0.14	0.39
	PRISm vs 0	-0.602 (0.45)	0.18	0.46
IGG3 CDR3 Length	age	0.012 (0.026)	0.65	0.87
	sex	0.62 (0.375)	0.1	0.31
	race	0.503 (0.484)	0.3	0.6
	dual use	1.338 (0.599)	0.027	0.14
	smoking	0.398 (0.628)	0.53	0.8
	vaping	0.644 (0.618)	0.3	0.6
	GOLD 1 vs 0	-1.065 (0.704)	0.13	0.37
	GOLD 2–4 vs 0	-0.796 (0.464)	0.088	0.28
	PRISm vs 0	-0.497 (0.761)	0.51	0.79
IGM CDR3 Length	age	-0.013 (0.013)	0.32	0.62
	sex	-0.569 (0.187)	0.0026	0.034
	race	0.658 (0.241)	0.0069	0.062
	dual use	0.177 (0.297)	0.55	0.8
	smoking	0.1 (0.311)	0.75	0.89
	vaping	-0.149 (0.306)	0.63	0.86
	GOLD 1 vs 0	0.051 (0.351)	0.89	0.97
	GOLD 2–4 vs 0	-0.329 (0.231)	0.16	0.41
	PRISm vs 0	0.134 (0.38)	0.72	0.87

CDR3 length is the length of the CDR3 sequence in nucleotides. Sex coded as (0=female, 1=male). Race coded as (0=self-reported Black race, 1=self-reported White race). PrISM, Preserved ratio impaired spirometry.

**Table 9:** Significant BCR associations to vaping, smoking, and dual use adjusting for socioeconomic status.

BCR measure	Dual Use			Cigarette smoking			Vaping		
	beta (se)	P-value	q-value	beta (se)	P-value	q-value	beta (se)	P-value	q-value
IgA1 expression	0.591 (0.185)	1.65E-03	2.97E-02	0.463 (0.197)	1.93E-02	1.25E-01	0.217 (0.19)	2.55E-01	5.79E-01
IgA2 expression	0.751 (0.207)	3.67E-04	1.28E-02	0.565 (0.219)	1.07E-02	9.01E-02	0.441 (0.212)	3.88E-02	1.84E-01
IgM expression	-0.448 (0.137)	1.27E-03	2.47E-02	-0.134 (0.146)	3.57E-01	6.80E-01	-0.295 (0.141)	3.76E-02	1.84E-01
IgA1 usage	0.079 (0.017)	6.01E-06	3.79E-04	0.047 (0.018)	9.80E-03	8.51E-02	0.037 (0.017)	3.67E-02	1.84E-01
IgA2 usage	0.042 (0.009)	2.32E-06	2.52E-04	0.024 (0.009)	9.14E-03	8.51E-02	0.023 (0.009)	8.63E-03	8.51E-02
IgG1 usage	0.024 (0.007)	8.14E-04	1.87E-02	0.016 (0.008)	4.03E-02	1.88E-01	0.017 (0.007)	2.30E-02	1.32E-01
IgG2 usage	0.013 (0.004)	2.95E-03	4.64E-02	0.005 (0.005)	2.25E-01	5.40E-01	0.005 (0.004)	2.18E-01	5.40E-01
IgM usage	-0.158 (0.031)	6.97E-07	1.76E-04	-0.096 (0.033)	3.84E-03	5.10E-02	-0.083 (0.032)	9.49E-03	8.51E-02
Class switching	0.119 (0.027)	2.22E-05	1.12E-03	0.064 (0.029)	2.85E-02	1.47E-01	0.052 (0.028)	6.61E-02	2.69E-01
IGHV1-18*01 class switching	0.005 (0.001)	4.60E-04	1.28E-02	0.004 (0.002)	1.47E-02	1.05E-01	0.004 (0.002)	1.38E-02	1.03E-01
IGHV3-7*01 class switching	0.006 (0.002)	4.97E-04	1.28E-02	0.003 (0.002)	1.10E-01	3.51E-01	0.002 (0.002)	3.65E-01	6.80E-01
IGHV5-51*01 class switching	0.009 (0.002)	8.26E-05	3.47E-03	0.005 (0.002)	2.46E-02	1.35E-01	0.006 (0.002)	1.21E-02	9.51E-02
IgE CDR3 Length	0.902 (2.986)	7.63E-01	8.95E-01	1.796 (3.159)	5.71E-01	8.22E-01	8.934 (3.242)	6.97E-03	7.64E-02

Results from regression model with the following covariates - age, race, sex, smoking/vaping status, GOLD stage, cigarette pack-years, income level, and regional socioeconomic deprivation index. Count values are log2 of unique BCR RNA sequence count. Usage is the proportion of all BCR RNA sequences falling into either a specific isotype or V allele category (calculated separately for isotypes and V alleles). Class switching proportion is the proportion of all BCR RNA sequences that belong to IgA, IgG, or IgE isotypes and have evidence of somatic hypermutation (>1 mutation relative to the IMGT reference database). CDR3 length is the length of the CDR3 sequence in nucleotides.

**Figure 4:**
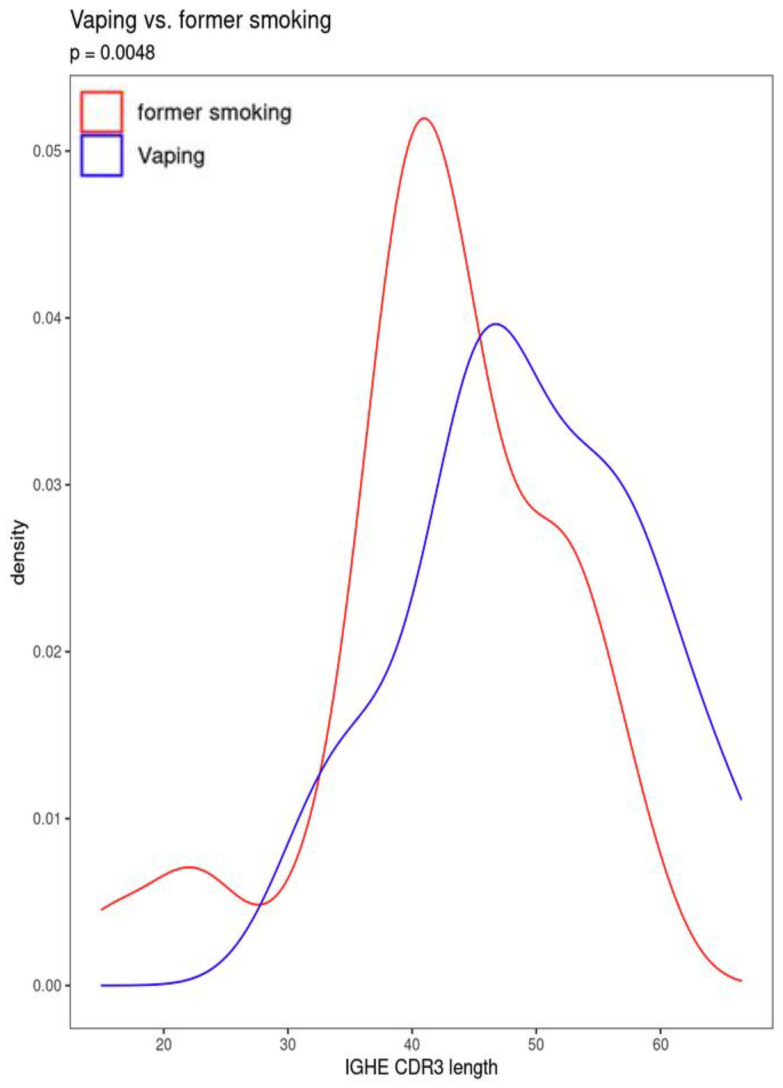
Difference in CDR3 length between current vaping and former smoking participants.

Current CS also was significantly associated with increased secretory IgA usage and decreased IgM usage, with several other suggestive associations. Vaping showed the same trend towards increased IgA usage and decreased IgM, though these associations were suggestive but not significant. There was also a suggestive association to decreased CDR3 length in IgE antibody transcripts (**Figure 4**). Overall, vaping and CS showed a similar trend in effect sizes compared to dual-use, suggesting that the effects were similar but less pronounced in current smokers and vapers relative to dual-users.

To examine the appropriateness of using former rather than never smokers as the reference group, we performed multivariable linear regressions comparing isotype usage in former versus never smoking individuals (**Table 10**), which demonstrated no significant differences between these groups after adjusting for multiple comparisons, suggesting that most of the smoking effects on B-cell function assessed in this study may resolve after cessation.

**Table 10:** BCR-seq associations comparing never to former smokers.

BCR-seq measure	beta	se	p-value
IgA1 expression	0.0077	0.2155	9.71E-01
IgA2 expression	-0.0443	0.2413	8.54E-01
IgD expression	0.0747	0.1775	6.74E-01
IgE expression	0.3765	0.3580	2.95E-01
IgG1 expression	0.0491	0.1839	7.90E-01
IgG2 expression	-0.1459	0.1891	4.41E-01
IgG3 expression	-0.2365	0.2085	2.58E-01
IgM expression	-0.0201	0.1612	9.01E-01
IgA1 usage	0.0016	0.0199	9.35E-01
IgA2 usage	0.0015	0.0101	8.83E-01
IgD usage	0.0073	0.0129	5.72E-01
IgE usage	0.0003	0.0002	1.42E-01
IgG1 usage	0.0017	0.0083	8.36E-01
IgG2 usage	-0.0025	0.0049	6.11E-01
IgG3 usage	-0.0020	0.0016	2.13E-01
IgM usage	-0.0098	0.0364	7.87E-01
Class switching	0.0079	0.0320	8.06E-01
IGHV1-18*01 class switching	0.0001	0.0017	9.67E-01
IGHV3-7*01 class switching	-0.0018	0.0019	3.54E-01
IGHV5-51*01 class switching	0.0013	0.0026	6.07E-01
IgA1 CDR3 Length	-0.4876	0.2728	7.53E-02
IgA2 CDR3 Length	0.1538	0.3645	6.73E-01
IgD CDR3 Length	0.0999	0.3773	7.91E-01
IgE CDR3 Length	10.7226	4.0377	9.10E-03
IgG1 CDR3 Length	0.0898	0.3679	8.07E-01
IgG2 CDR3 Length	-0.0272	0.4200	9.48E-01
IgG3 CDR3 Length	1.2093	0.7119	9.08E-02
IgM CDR3 Length	0.2856	0.3545	4.21E-01

Count values are log2 of unique BCR RNA sequence count. Usage is the proportion of all BCR RNA sequences falling into either a specific isotype or V allele category (calculated separately for isotypes and V alleles). Class switching proportion is the proportion of all BCR RNA sequences that belong to IgA, IgG, or IgE isotypes and have evidence of somatic hypermutation (>1 mutation relative to the IMGT reference database). CDR3 length is the length of the CDR3 sequence in nucleotides.

### Associations between sex and self-reported race on antibody production

**Figure 5:**
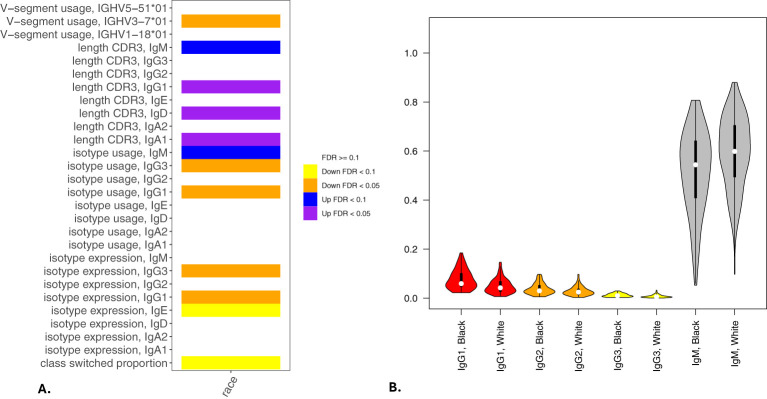
Associations of BCR measures with self-reported race. Significant associations between BCR measures and self-reported AA or NHW race from multivariable models analyzing class switching, isotype expression and usage, V allele usage, and CDR3 length (in nucleotides) are shown in **(A)**. **(B)** shows IgG and IgM isotype usage by self-reported race. Significance is assessed by t-tests. *p<=0.05, **p<=0.005, ns p>0.05.

**Figure 6:**
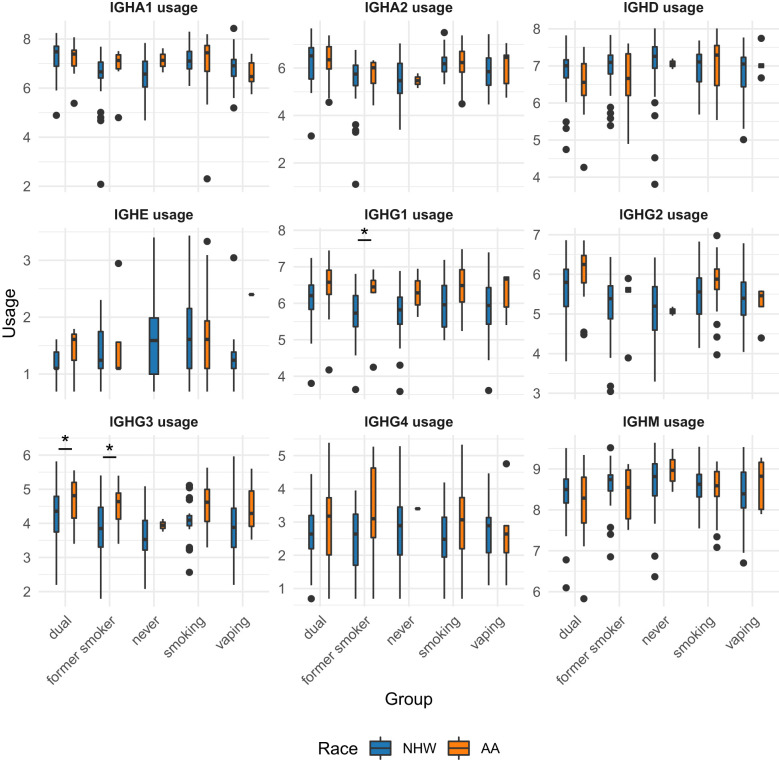
Univariate associations of immunoglobulin subtype usage across smoking/vaping group, separated by self-identified race. NHW, non-Hispanic white; AA, African American. Student t-tests were performed and p-values < 0.05 are indicate by with an asterisk (*).

In multivariable models, we observed significant associations for 8 BCR-seq measures with self-reported race (**Table 11**). Comparing NHW versus AA participants, NHW-identifying participants had decreased usage of IgG1 and IgG3 isotypes and increased usage of IgM (**Figure 5**). Univariate associations are shown in **Figure 6**. To investigate the extent to which these associations may be driven by variables related to income or socioeconomic status, we repeated the analysis adjusting for self-reported income level and area deprivation index, and 4 of the 8 significant associations remained significant, and all 8 associations had a consistent effect direction (**Table 12**). After adjusting for principal components of genetic similarity, the isotype usage associations with race were attenuated, though notably, the principal component variables were also not associated with isotype usage. We observed no interaction between self-identified race and CS, vaping, or dual-use on isotype usage (all p > 0.05). In the sex analysis, we observed one significant association in which male compared to female sex was significantly associated with decreased CDR3 length in IgM isotype sequences (p < 0.05).

**Table 11:** Significant BCR associations to self-reported race.

BCR Measure	Self-reported AA vs NHW Participants		
	β (se)	P-value	q-value
IgG1 CDR3 Length	1.273 (0.25)	7.91e-07	1.99E-04
IgG1 usage	-0.025 (0.006)	1.16e-05	5.84E-04
IgG3 usage	-0.004 (0.001)	1.34E-04	4.22E-03
IgD CDR3 Length	0.914 (0.257)	4.57E-04	1.03E-02
IgG3 expression	-0.5 (0.143)	5.57E-04	1.08E-02
IgA1 CDR3 Length	0.645 (0.186)	6.15E-04	1.11E-02
IGHV3-7*01 class switching	-0.004 (0.001)	1.83E-03	2.89E-02
IgG1 expression	-0.384 (0.126)	2.56E-03	3.43E-02
Class switching	-0.059 (0.022)	6.96E-03	6.15E-02
IgM CDR3 Length	0.658 (0.241)	6.88E-03	6.15E-02
IgE expression	-0.569 (0.215)	9.28E-03	7.36E-02
IgM usage	0.063 (0.025)	1.14E-02	8.22E-02

Count values are log2 of unique BCR RNA sequence count. Usage is the proportion of all BCR RNA sequences falling into either a specific isotype or V allele category (calculated separately for isotypes and V alleles). Class switching proportion is the proportion of all BCR RNA sequences that belong to IgA, IgG, or IgE isotypes and have evidence of somatic hypermutation (>1 mutation relative to the IMGT reference database). CDR3 length is the length of the CDR3 sequence in nucleotides. NHW is the reference group (i.e. negative β means lower in NHW).

**Table 12:** BCR-seq associations to self-reported race adjusting for income level and national area deprivation index.

BCR-seq measure	beta (se)	p-value	q-value
IgA1 expression	-0.085 (0.158)	5.92E-01	8.22E-01
IgA2 expression	-0.097 (0.178)	5.87E-01	8.22E-01
IgD expression	0.024 (0.131)	8.53E-01	9.27E-01
IgE expression	-0.371 (0.233)	1.14E-01	3.55E-01
IgG1 expression	-0.346 (0.136)	1.19E-02	9.51E-02
IgG2 expression	-0.222 (0.142)	1.20E-01	3.64E-01
IgG3 expression	-0.447 (0.156)	4.60E-03	5.51E-02
IgM expression	0.119 (0.117)	3.09E-01	6.24E-01
IgA1 usage	-0.007 (0.015)	6.52E-01	8.40E-01
IgA2 usage	-0.003 (0.007)	6.84E-01	8.57E-01
IgD usage	-0.005 (0.01)	5.90E-01	8.22E-01
IgE usage	0 (0)	2.01E-01	5.35E-01
IgG1 usage	-0.021 (0.006)	5.07E-04	1.28E-02
IgG2 usage	-0.006 (0.004)	1.08E-01	3.49E-01
IgG3 usage	-0.004 (0.001)	3.13E-03	4.64E-02
IgM usage	0.06 (0.026)	2.43E-02	1.35E-01
Class switching	-0.053 (0.023)	2.53E-02	1.35E-01
IGHV1-18*01 class switching	-0.002 (0.001)	8.45E-02	3.06E-01
IGHV3-7*01 class switching	-0.004 (0.001)	9.59E-03	8.51E-02
IGHV5-51*01 class switching	0 (0.002)	8.19E-01	9.15E-01
IgA1 CDR3 Length	0.584 (0.202)	4.21E-03	5.30E-02
IgA2 CDR3 Length	-0.157 (0.27)	5.60E-01	8.20E-01
IgD CDR3 Length	0.926 (0.278)	1.02E-03	2.15E-02
IgE CDR3 Length	-1.73 (2.572)	5.03E-01	7.63E-01
IgG1 CDR3 Length	1.305 (0.272)	3.00E-06	2.52E-04
IgG2 CDR3 Length	0.155 (0.307)	6.14E-01	8.32E-01
IgG3 CDR3 Length	0.621 (0.524)	2.37E-01	5.51E-01
IgM CDR3 Length	0.588 (0.263)	2.64E-02	1.38E-01

Count values are log2 of unique BCR RNA sequence count. Usage is the proportion of all BCR RNA sequences falling into either a specific isotype or V allele category (calculated separately for isotypes and V alleles). Class switching proportion is the proportion of all BCR RNA sequences that belong to IgA, IgG, or IgE isotypes and have evidence of somatic hypermutation (>1 mutation relative to the IMGT reference database). CDR3 length is the length of the CDR3 sequence in nucleotides.

### Associations to COPD and related phenotypes

We also examined associations between BCR-seq measures and age, COPD affection status, and CT-related measures of emphysema and airway wall thickness. We observed a significant univariate association between COPD and increased usage of the IGHV5-51*01 V allele and suggestive associations with increased class switching and a shift from IgM to IgA (**Table 13**). Similar but non-significant trends were associated with the CT-quantified airway wall thickness (**Table 14**). However, these associations were not significant in models adjusting for CS/vaping behavior, age, sex, and race. No significant associations were observed with CT emphysema measures.

**Table 13:** Significant "univariate" associations to COPD.

Dependent variable	beta_se	P-value	q-value
IgA2 usage	0.022 (0.006)	2.99E-04	1.28E-02
IGHV5-51*01 class switching	0.005 (0.001)	3.06E-04	1.28E-02
IgA1 usage	0.04 (0.012)	5.79E-04	1.62E-02
IgM usage	-0.071 (0.022)	1.22E-03	2.55E-02
IgM expression	-0.272 (0.091)	3.29E-03	5.54E-02
Class switching	0.054 (0.019)	4.91E-03	6.87E-02
IgG3 usage	0.002 (0.001)	1.58E-02	1.73E-01
IgG2 usage	0.006 (0.003)	2.66E-02	2.42E-01
IgD expression	-0.216 (0.098)	2.88E-02	2.42E-01
IgD CDR3 Length	-0.44 (0.215)	4.17E-02	2.86E-01
IgM CDR3 Length	-0.407 (0.2)	4.34E-02	2.86E-01
IGHV1-18*01 class switching	0.002 (0.001)	4.42E-02	2.86E-01
IgA2 expression	0.265 (0.14)	5.93E-02	3.52E-01
IgA1 expression	0.201 (0.124)	1.06E-01	4.93E-01
IgG2 CDR3 Length	-0.362 (0.23)	1.17E-01	5.09E-01
IgG3 CDR3 Length	-0.617 (0.397)	1.21E-01	5.09E-01
IgG1 usage	0.007 (0.005)	1.36E-01	5.16E-01
IgE usage	0 (0)	1.41E-01	5.16E-01
IgA2 CDR3 Length	0.292 (0.201)	1.48E-01	5.17E-01
IgE CDR3 Length	-2.312 (1.929)	2.33E-01	5.96E-01
IgD usage	-0.008 (0.007)	2.35E-01	5.96E-01
IgG3 expression	0.137 (0.124)	2.71E-01	6.15E-01
IgG1 CDR3 Length	-0.216 (0.215)	3.15E-01	6.79E-01
IGHV3-7*01 class switching	0.001 (0.001)	3.42E-01	6.86E-01
IgG2 expression	0.101 (0.109)	3.52E-01	6.88E-01
IgA1 CDR3 Length	0.131 (0.154)	3.95E-01	7.29E-01
IgE expression	0.149 (0.176)	3.99E-01	7.29E-01
IgG1 expression	-0.01 (0.108)	9.26E-01	9.59E-01

Associations are from models with the BCR measure as the response adjusting for only GOLD spirometric stage and inhaled corticosteroid use. Count values are log2 of unique BCR RNA sequence count. GOLD stage coded as GOLD 2-4, GOLD 1, and PrISM with GOLD 0 as the reference. Usage is the proportion of all BCR RNA sequences falling into either a specific isotype or V allele category (calculated separately for isotypes and V alleles). Class switching proportion is the proportion of all BCR RNA sequences that belong to IgA, IgG, or IgE isotypes and have evidence of somatic hypermutation (>1 mutation relative to the IMGT reference database).

**Table 14:** Significant "univariate" associations to airway wall thickness.

Dependent variable	beta_se	P-value	q-value
IGHV5-51*01 class switching	0.00027 (9e-05)	1.95E-03	5.46E-02
IgM CDR3 Length	-0.03027 (0.01064)	4.88E-03	6.83E-02
IgD CDR3 Length	-0.03093 (0.01156)	8.04E-03	7.50E-02
IgM expression	-0.01258 (0.00502)	1.29E-02	9.03E-02
IgD expression	-0.01083 (0.00532)	4.29E-02	2.23E-01
IgM usage	-0.00245 (0.00123)	4.77E-02	2.23E-01
IgA1 usage	0.00126 (0.00067)	6.04E-02	2.42E-01
IgG3 usage	9e-05 (5e-05)	7.92E-02	2.52E-01
Class switching	0.00187 (0.00106)	8.10E-02	2.52E-01
IgG1 usage	4e-04 (0.00027)	1.41E-01	3.86E-01
IgA1 expression	0.00959 (0.0068)	1.60E-01	3.86E-01
IgE usage	1e-05 (1e-05)	1.66E-01	3.86E-01
IgE expression	0.01106 (0.00986)	2.64E-01	5.43E-01
IGHV1-18*01 class switching	6e-05 (6e-05)	3.08E-01	5.43E-01
IgG2 usage	0.00016 (0.00016)	3.10E-01	5.43E-01
IgA2 usage	0.00034 (0.00034)	3.10E-01	5.43E-01
IgA2 expression	0.00648 (0.00785)	4.10E-01	6.75E-01
IgG3 expression	0.00359 (0.00673)	5.95E-01	8.11E-01
IgG2 expression	0.00299 (0.0059)	6.13E-01	8.11E-01
IgG1 expression	-0.00289 (0.00572)	6.14E-01	8.11E-01
IgG1 CDR3 Length	-0.00548 (0.01166)	6.39E-01	8.11E-01
IgE CDR3 Length	-0.05574 (0.12016)	6.44E-01	8.11E-01
IgD usage	-0.00017 (4e-04)	6.66E-01	8.11E-01
IgG3 CDR3 Length	-0.00522 (0.02196)	8.12E-01	9.48E-01
IgA1 CDR3 Length	0.00155 (0.00831)	8.52E-01	9.54E-01
IgG2 CDR3 Length	0.00053 (0.01308)	9.67E-01	9.94E-01
IGHV3-7*01 class switching	0 (6e-05)	9.80E-01	9.94E-01
IgA2 CDR3 Length	8e-05 (0.01106)	9.94E-01	9.94E-01

Associations are from models with the BCR measure as the response adjusting for only Wall Area Thickness, CT scanner model, and inhaled corticosteroid use. Wall area thickness % is measured as the average thickness of segmental airway walls / total airway area. Count values are log2 of unique BCR RNA sequence count. Usage is the proportion of all BCR RNA sequences falling into either a specific isotype or V allele category (calculated separately for isotypes and V alleles).

## Discussion

In this study of 234 individuals with B-cell receptor sequencing (BCR-seq) and cigarette smoking (CS), vaping, and COPD-related outcome data, we observed significant effects of CS and vaping leading to increased IgA expression and usage, increased class switching, and lower antibody diversity indicating greater clonal activation of specific B-cell populations. Taken together, these results demonstrate the potential dangers of dual-use compared to single product use, an under-recognized but important public health concern.

Our study demonstrates that dual use and CS are associated increased B-cell activation and increased production of secretory IgA (i.e. IgA2), consistent with mucosal exposure to compounds from vaping devices and combustible cigarettes. We note that vaping also showed a similar but nonsignificant trend with a consistent effect direction. It is possible that in a larger study these associations would reach statistical significance. IgA is secreted by the airway mucosa and is important in lung immune defense against pathogens (20). Our data suggest that CS and vaping trigger similar host immune responses with a greater effect observed in participants engaged in dual-use. Indeed, many patients who use e-cigarettes for smoking cessation will smoke tobacco cigarettes and vape, and our results underscore the importance of understanding the health effects of dual-use specifically, as well as vaping and CS alone.

Our findings are consistent with previous research demonstrating that CS increases IgA production in blood and lung (21, 22). Higher levels of class-switched memory B-cells have been observed in individuals who smoke compared to former and never smokers, irrespective of COPD status (23). Vaping is associated with increased circulating club cell protein and decreased transcutaneous oxygen tension (3), increased IL-10 and TNF-*α* (24), and methylation changes that may cause long-term alterations in cytokine levels (25, 26). Vaping has also been associated with increased expression of 191 inflammatory proteins from bronchoalveolar lavage fluid, including MUC5AC, which is important in mucin production (27). Clinically, vaping is associated with acute lung injury (2, 28), decreased FEV_1_/FVC and peak expiratory flow in asthmatics (24), and chronic bronchitis (27). However, the implications of dual-use on adaptive immunity are an important contribution of our study.

There are several mechanistic questions raised by our finding that dual-use leads to greater B-cell activation and class switching. The toxicology of cigarette smoking is highly complex and heterogeneous, involving thousands of compounds with variable delivery and dosage influenced by numerous factors such as solvents, filters, tobacco content, burn time, and individual inhalation patterns. The toxicology of vaping is less understood but shares many of the same intricate considerations. Nonetheless, a wealth of literature on the effects of smoking and vaping allow us to hypothesize on how smoking and vaping might be altering B-cell function. CS contains benzo-a-pyrene, a potent activator of aryl-hydrocarbon receptor. AHR is expressed in B-cell developmental subsets upon activation and is associated with a suppressed humoral immune response (29). Whether this mechanism can explain our findings for CS and/or vaping remains to be seen but is worth further investigation. Nitrosamines are metabolic products of nicotine and present in the saliva of both individuals who smoke cigarettes or vape. Nitrosamines can cause DNA adducts and induce formation of reactive oxygen species and NFkB-driven inflammation (30), the latter of which is an important regulator of B-cell differentiation and activation (31). Both smoking and vaping can lead to the release of damage-associated molecular patterns (DAMPs) and pathogen-associated molecular patterns (PAMPs) that can activate antigen-presenting cells that stimulate B-cell activation. Several other potential explanations and mechanistic pathways can be explored through further studies based on our findings.

The role of adaptive immunity and B-cells in COPD and emphysema pathogenesis is well-recognized (10, 20, 32, 33). Although we observed several univariable associations of blood B-cell transcriptomics to COPD and related phenotypes, none of these associations remained significant in multivariable models after adjustment for multiple comparisons. While seemingly in contrast to the well-known increase in lung lymphoid follicles and B-cell infiltration in severe COPD (10–12, 32, 33), this lack of significant associations is not surprising, as by definition the presence of germinal centers in lung lymphoid follicles indicates local B-cell division and maturation. Indeed, antigen exposure within the lung leads to local recruitment of memory (and perhaps naïve) B-cells, local expansion of B-cells and plasma cells within the airway tissue, and subsequent production of antigen-specific antibodies that tend to stay in the lung before getting into the bloodstream (10, 34). Since our analysis is limited to the transcriptome of circulating B-cells, we cannot address whether there is protein-level spillover of antibody produced by lung-resident B-cells into the blood, though other COPD-specific inflammatory changes in blood such as neutrophilia and the increase in several RNA and protein biomarkers are well-documented (35–37).

An intriguing aspect of BCR-seq is the ability to characterize the B-cell response at the level of specific V alleles. IGHV5-51*01 usage was increased in dual users, and as this V allele is increased in response to parainfluenza infections, it might suggest that these individuals are more susceptible to infections or are experiencing immunological reactions similar to those invoked by this viral infection. There was a univariate association of IGHV5-51*01 with COPD status, which might suggest that this V segment could link immune responses of smoking and vaping with COPD pathogenesis. IGHV1-18*01 has been associated with chronic lymphocytic leukemia (38). There is no known immunologic link between dual use and chronic lymphocytic leukemia, though such a connection would be of public health importance. The relevance of IGHV3-1*01 is less clear though this association is interesting and can be investigated in future studies.

We selected former smokers as the reference group because the vast majority of participants in our cohort were either current or former smokers, and vaping in this population typically occurs in the context of prior tobacco use. However, we acknowledge that the persistence of memory B-cell responses from prior smoking may confound the interpretation of our findings. This limitation highlights the importance of future studies that include a larger never-smoking comparator group to better isolate the effects of vaping and dual use.

Our sequencing strategy, which relied on forward primers targeting V genes in the variable region, carries a risk of primer binding bias due to mutations in the primer binding sites, particularly in groups with higher levels of B-cell activation. While this bias is somewhat mitigated because all participant groups were subjected to the same methodology, the unequal levels of B-cell activation between groups may have introduced skewed results, and future studies using template-switching approaches could help address this limitation.

To our knowledge, this is the largest study to date of BCR-seq in humans or model systems. The strengths of this study include the novel use of BCR-seq in a large, deeply phenotyped cohort of participants engaged in current CS, vaping, or both. One limitation is that we did not have a suitable replication cohort, but our current findings highlight the need to obtain BCR-seq data longitudinally and in additional cohorts. We were not able to compare B-cell activation in lung versus blood, which is important for understanding the role of adaptive immunity in CS, vaping, and COPD pathogenesis. Single-cell and spatial transcriptomic or proteomic data would provide greater resolution of the adaptive immune responses to CS and vaping. T cell receptor sequencing that coincides with BCR-seq would provide a more comprehensive view of adaptive immune responses in this context as well. Ideally, we would have measured immunoglobulin protein levels concomitantly with BCR sequencing. However, BCR-seq has been used to analyze B-cell activation independently of circulating protein levels, as demonstrated in a study of meningococcal vaccine responses, where sequencing revealed distinct plasma cell signatures and vaccine-induced repertoire shifts that would not be captured by traditional immunogenicity measures (39). A larger sample size would be desirable to examine COPD-related outcomes, particularly longitudinal outcomes such as FEV_1_ decline, mortality, and exacerbations. Self-identified race was a secondary outcome, and this cohort recruited only non-Hispanic white and African American individuals, which is not ideal for this type of analysis. Ideally, a more diverse sample representative of additional racial and ethnic groups would be included, and this limitation suggests caution in interpreting the race associations reported in this study.

In conclusion, we observed that CS and vaping each enhance B-cell activation, and that dual-users show a trend towards greater effects than either alone. Self-identified race was strongly associated with IgG isotype usage. These findings highlight associations between B-cell activation and antibody transcription, suggesting potential differences in immune and vaccine responses linked to CS, vaping, and self-identified race.

## Data Availability

The datasets presented in this study can be found in online repositories. The names of the repository/repositories and accession number(s) can be found below: https://www.ncbi.nlm.nih.gov/gap/, phs000951.v6.p5.
