# Supplementary material for: B-cell immune repertoire sequencing in tobacco cigarette smoking, vaping, and chronic obstructive pulmonary disease in the COPDGene cohort
Source: Front Immunol. 2025 May 2;16:1508786. doi: 10.3389/fimmu.2025.1508786 (PMC12082042; doi:10.3389/fimmu.2025.1508786)
Supplement: Supplementary file 1 [file DataSheet1.pdf]

Supplement to "B cell immune repertoire sequencing in smoking, vaping, and chronic obstructive pulmonary disease in the COPDGene cohort"

Funding and Acknowledgements 2

Supplementary Methods 5

B cell receptor repertoire sequencing (BCR-seq) library preparation 5

AIRR-seq data processing 5

Supplementary Discussion 6

References 7

Funding and Acknowledgements

COPDGene Phase 3 Grant Support and Disclaimer

The COPDGene study (NCT00608764) is supported by grants from the NHLBI (U01HL089897 and U01HL089856) and by NIH contract 75N92023D00011. The content is solely the responsibility of the authors and does not necessarily represent the official views of the National Heart, Lung, and Blood Institute or the National Institutes of Health.

COPD Foundation Funding

COPDGene is also supported by the COPD Foundation through contributions made to an Industry Advisory Board that has included AstraZeneca, Bayer Pharmaceuticals, Boehringer- Ingelheim, Genentech, GlaxoSmithKline, Novartis, Pfizer, and Sunovion.

COPDGene® Investigators – Core Units

Administrative Center: James D. Crapo, MD (PI); Edwin K. Silverman, MD, PhD (PI); Barry J. Make, MD; Elizabeth A. Regan, MD, PhD

Genetic Analysis Center: Terri H. Beaty, PhD; Peter J. Castaldi, MD, MSc; Michael H. Cho, MD, MPH; Dawn L. DeMeo, MD, MPH; Adel El Boueiz, MD, MMSc; Marilyn G. Foreman, MD, MS; Auyon Ghosh, MD; Lystra P. Hayden, MD, MMSc; Craig P. Hersh, MD, MPH; Jacqueline Hetmanski, MS; Brian D. Hobbs, MD, MMSc; John E. Hokanson, MPH, PhD; Wonji Kim, PhD; Nan Laird, PhD; Christoph Lange, PhD; Sharon M. Lutz, PhD; Merry-Lynn McDonald, PhD; Dmitry Prokopenko, PhD; Matthew Moll, MD, MPH; Jarrett Morrow, PhD; Dandi Qiao, PhD; Elizabeth A. Regan, MD, PhD; Aabida Saferali, PhD; Phuwanat Sakornsakolpat, MD; Edwin K. Silverman, MD, PhD; Emily S. Wan, MD; Jeong Yun, MD, MPH

Imaging Center: Juan Pablo Centeno; Jean-Paul Charbonnier, PhD; Harvey O. Coxson, PhD; Craig J. Galban, PhD; MeiLan K. Han, MD, MS; Eric A. Hoffman, Stephen Humphries, PhD; Francine L. Jacobson, MD, MPH; Philip F. Judy, PhD; Ella A. Kazerooni, MD; Alex Kluiber; David A. Lynch, MB; Pietro Nardelli, PhD; John D. Newell, Jr., MD; Aleena Notary; Andrea Oh, MD; Elizabeth A. Regan, MD, PhD; James C. Ross, PhD; Raul San Jose Estepar, PhD; Joyce Schroeder, MD; Jered Sieren; Berend C. Stoel, PhD; Juerg Tschirren, PhD; Edwin Van Beek, MD, PhD; Bram van Ginneken, PhD; Eva van Rikxoort, PhD; Gonzalo Vegas Sanchez- Ferrero, PhD; Lucas Veitel; George R. Washko, MD; Carla G. Wilson, MS;

PFT QA Center, Salt Lake City, UT: Robert Jensen, PhD

Data Coordinating Center and Biostatistics, *National Jewish Health, Denver, CO*: Douglas Everett, PhD; Jim Crooks, PhD; Katherine Pratte, PhD; Matt Strand, PhD; Carla G. Wilson, MS

Epidemiology Core, *University of Colorado Anschutz Medical Campus, Aurora, CO*: John E. Hokanson, MPH, PhD; Erin Austin, PhD; Gregory Kinney, MPH, PhD; Sharon M. Lutz, PhD; Kendra A. Young, PhD

Mortality Adjudication Core: Surya P. Bhatt, MD; Jessica Bon, MD; Alejandro A. Diaz, MD, MPH; MeiLan K. Han, MD, MS; Barry Make, MD; Susan Murray, ScD; Elizabeth Regan, MD; Xavier Soler, MD; Carla G. Wilson, MS

Biomarker Core: Russell P. Bowler, MD, PhD; Katerina Kechris, PhD; Farnoush Banaei- Kashani, PhD

COPDGene® Investigators – Clinical Centers

Ann Arbor VA: Jeffrey L. Curtis, MD; Perry G. Pernicano, MD

Baylor College of Medicine, Houston, TX: Nicola Hanania, MD, MS; Mustafa Atik, MD; Aladin Boriek, PhD; Kalpatha Guntupalli, MD; Elizabeth Guy, MD; Amit Parulekar, MD;

Brigham and Women's Hospital, Boston, MA: Dawn L. DeMeo, MD, MPH; Craig Hersh, MD, MPH; Francine L. Jacobson, MD, MPH; George Washko, MD

Columbia University, New York, NY: R. Graham Barr, MD, DrPH; John Austin, MD; Belinda D'Souza, MD; Byron Thomashow, MD

Duke University Medical Center, Durham, NC: Neil MacIntyre, Jr., MD; H. Page McAdams, MD; Lacey Washington, MD

HealthPartners Research Institute, Minneapolis, MN: Charlene McEvoy, MD, MPH; Joseph Tashjian, MD

Johns Hopkins University, Baltimore, MD: Robert Wise, MD; Robert Brown, MD; Nadia N. Hansel, MD, MPH; Karen Horton, MD; Allison Lambert, MD, MHS; Nirupama Putcha, MD, MHS

Lundquist Institute for Biomedical Innovation at Harbor UCLA Medical Center, Torrance, CA: Richard Casaburi, PhD, MD; Alessandra Adami, PhD; Matthew Budoff, MD; Hans Fischer, MD; Janos Porszasz, MD, PhD; Harry Rossiter, PhD; William Stringer, MD

Michael E. DeBakey VAMC, Houston, *TX*: Amir Sharafkhaneh, MD, PhD; Charlie Lan, DO *Minneapolis VA:* Christine Wendt, MD; Brian Bell, MD; Ken M. Kunisaki, MD, MS

Morehouse School of Medicine, Atlanta, GA: Eric L. Flenaugh, MD; Hirut Gebrekristos, PhD; Mario Ponce, MD; Silanath Terpenning, MD; Gloria Westney, MD, MS

National Jewish Health, Denver, CO: Russell Bowler, MD, PhD; David A. Lynch, MB *Reliant Medical Group, Worcester, MA*: Richard Rosiello, MD; David Pace, MD

Temple University, Philadelphia, PA: Gerard Criner, MD; David Ciccolella, MD; Francis Cordova, MD; Chandra Dass, MD; Gilbert D'Alonzo, DO; Parag Desai, MD; Michael Jacobs, PharmD; Steven Kelsen, MD, PhD; Victor Kim, MD; A. James Mamary, MD; Nathaniel Marchetti, DO; Aditi Satti, MD; Kartik Shenoy, MD; Robert M. Steiner, MD; Alex Swift, MD; Irene Swift, MD; Maria Elena Vega-Sanchez, MD

University of Alabama, Birmingham, AL: Mark Dransfield, MD; William Bailey, MD; Surya P. Bhatt, MD; Anand Iyer, MD; Hrudaya Nath, MD; J. Michael Wells, MD

University of California, San Diego, CA: Douglas Conrad, MD; Xavier Soler, MD, PhD; Andrew Yen, MD

University of Iowa, Iowa City, IA: Alejandro P. Comellas, MD; Karin F. Hoth, PhD; John Newell, Jr., MD; Brad Thompson, MD

University of Michigan, Ann Arbor, MI: MeiLan K. Han, MD MS; Ella Kazerooni, MD MS; Wassim Labaki, MD MS; Craig Galban, PhD; Dharshan Vummidi, MD

University of Minnesota, Minneapolis, MN: Joanne Billings, MD; Abbie Begnaud, MD; Tadashi Allen, MD

University of Pittsburgh, Pittsburgh, PA: Frank Sciurba, MD; Jessica Bon, MD; Divay Chandra, MD, MSc; Joel Weissfeld, MD, MPH

University of Texas Health, San Antonio, San Antonio, TX: Antonio Anzueto, MD; Sandra Adams, MD; Diego Maselli-Caceres, MD; Mario E. Ruiz, MD; Harjinder Singh

Supplementary Methods

B cell receptor repertoire sequencing (BCR-seq) library preparation

Library preparation was performed according to Vollmers et al.[1]. Briefly, a pooled set of 5 isotype-specific IGH constant region primers (RT_pool), containing 12 random nucelotides (nt) and partial Illumina adapters were added to the RNA samples, incubated at 72°C for 3 min then immediately placed on ice for 2 min. First-strand cDNA synthesis was then performed using Smartscribe reverse transcriptase (Takara) according to manufacturer's instructions. Second-strand cDNA synthesis was done using Phusion HiFi DNA polymerase (ThermoFisher) using a pool of six IGH variable region primers (V_FR1_pool) and a pool of five IGH constant region primers (C_pool), both containing 12 random nts and partial Illumina adapters. The reaction was incubated using the following heat cycle program: [98°C for 4 min, 52°C for 1 min, 72°C for 5 min]x2. The resulting double-stranded cDNA was purified and size-selected for molecules > 350bp using Zymo Select-a-Size columns. Finally, the double-stranded cDNA was amplified using 2x kapa hifi hotstart readymix (Roche) using primers containing complete Illumina adapters indexes (NexteraA_Index and NexteraB_Index primers) and the following heat cycle program: 95C for 3 mins, [98C for 20s, 67C for 15s, 72C for 1min]x26, 72C for 5 mins. PCR products were purified once using Ampure XP beads at a 0.7:1 ratio then pooled for multiplexed sequencing using the Illumina MiSeq sequencer and 2x300 run kits and flowcells.

AIRR-seq data processing

We downloaded from IMGT the reference germline sequences for IGH V (IMGT-gapped), D and J genes and created blast database files following instructions from NCBI IgBLAST. Blast database for IGH C genes were directly downloaded from NCBI IgBLAST release ftp site. Fasta files of preprocessed AIRR-seq reads were then aligned to IGH V, D, J and C genes using igblastn command line program from IgBLAST (version 1.19.0). We further filtered aligned reads keeping only those with non-missing v_support and j_support fields, v_support >= 1e-50 and predicted to be productive. We used the spectralClones function from the scoper R package to infer clonal relationships between the sequence reads.

Supplementary Discussion

Our finding of higher levels of IgG1 and IgG3 in AA compared to NHW participants agrees with a previous report[2], which we extend by demonstrating this association in the context of smoking/vaping behaviors. However, there was no statistical interaction such that the combination of race and smoking/vaping variables demonstrated greater effects on isotype usage than the sum of their individual effects - though we note that our study is not well-powered to detect interactions of modest effect. Despite the lack of a large literature on racial differences in B-cell function, this topic is of substantial interest due to the increased risk of multiple myeloma in AA individuals[3]. IgG1 and IgG3 both have excellent complement activation and opsonization capabilities, and IgG3 is a potent immune effector, suggesting differences in response to pathogens and toxins. The degree to which our findings represent interactions between self-identified race and gene-by-environment interactions, specifically the effects of racism, are unclear.

References

1. Vollmers, C., Sit, R. V., Weinstein, J. A., Dekker, C. L. & Quake, S. R. Genetic measurement of memory B-cell recall using antibody repertoire sequencing. *Proc Natl Acad Sci U S A* **110**, 13463–13468 (2013).
2. Bunce, C. M. & Drayson, M. T. Dissecting racial disparities in multiple myeloma-clues from differential immunoglobulin levels. *Blood Cancer J* **10**, 44 (2020).
3. Bhutani, M. *et al.* Addressing the disparities: the approach to the African American patient with multiple myeloma. *Blood Cancer J* **13**, 189 (2023).
